# Quantum Diffie–Hellman Extended to Dynamic Quantum Group Key Agreement for e-Healthcare Multi-Agent Systems in Smart Cities

**DOI:** 10.3390/s20143940

**Published:** 2020-07-15

**Authors:** Vankamamidi S. Naresh, Moustafa M. Nasralla, Sivaranjani Reddi, Iván García-Magariño

**Affiliations:** 1 Department of Computer Science and Engineering, Sri Vasavi Engineering College, Tadepalligudeam 534101, India; vsnaresh111@gmail.com; 2 Department of Communications and Networks Engineering, College of Engineering, Prince Sultan University, Riyadh 11586, Saudi Arabia; mnasralla@psu.edu.sa; 3 Department of Computer Science and Engineering, Anil Neerukonda Institute of Technology & Science, Visakhapatnam 530003, India; rsivaranjani552008@gmail.com; 4 Department of Software Engineering and Artificial Intelligence, Faculty of Computer Science, Complutense University of Madrid, 28040 Madrid, Spain; 5 Instituto de Tecnología del Conocimiento, UCM, 28040 Madrid, Spain

**Keywords:** quantum group key, quantum summation, quantum information, quantum teleportation, participant attacks, sensor, multi-agent system

## Abstract

Multi-Agent Systems can support e-Healthcare applications for improving quality of life of citizens. In this direction, we propose a healthcare system architecture named smart healthcare city. First, we divide a given city into various zones and then we propose a zonal level three-layered system architecture. Further, for effectiveness we introduce a Multi-Agent System (MAS) in this three-layered architecture. Protecting sensitive health information of citizens is a major security concern. Group key agreement (GKA) is the corner stone for securely sharing the healthcare data among the healthcare stakeholders of the city. For establishing GKA, many efficient cryptosystems are available in the classical field. However, they are yet dependent on the supposition that some computational problems are infeasible. In light of quantum mechanics, a new field emerges to share a secret key among two or more members. The unbreakable and highly secure features of key agreement based on fundamental laws of physics allow us to propose a Quantum GKA (QGKA) technique based on renowned Quantum Diffie–Hellman (QDH). In this, a node acts as a Group Controller (GC) and forms 2-party groups with remaining nodes, establishing a QDH-style shared key per each two-party. It then joins these keys into a single group key by means of a XOR-operation, acting as a usual group node. Furthermore, we extend the QGKA to Dynamic QGKA (DQGKA) by adding join and leave protocol. Our protocol performance was compared with existing QGKA protocols in terms of Qubit efficiency (QE), unitary operation (UO), unitary operation efficiency (UOE), key consistency check (KCC), security against participants attack (SAP) and satisfactory results were obtained. The security analysis of the proposed technique is based on unconditional security of QDH. Moreover, it is secured against internal and external attack. In this way, e-healthcare Multi-Agent System can be robust against future quantum-based attacks.

## 1. Introduction

Nowadays the creation of a healthy society is the major concern, as the people living in this society are facing many problems—especially in healthcare. Over the years, advancement in the medical sciences has created effective diagnosis solutions to many life-threatening diseases. However, the rapid growth of technology, population and urban lifestyles have increased the demand to think about the smartness [1,2,3] of healthcare networks, in which the people will get the medical monitoring and treatment in a more quick and efficient manner.

In order to better provide healthcare services to the needy, healthcare stake holders such as citizens, medical practitioners, pharmaceutical companies, healthcare specialists, researchers and metropolitan managers are working together in an integrated Internet of Things (IoT)-environment in order to (i) offer emergency services with minimized healthcare access response time, (ii) offer remote treatment, (iii) collaborate with hospitals and doctors around the city and (iv) save time, money—and eventually lives.

E-healthcare technology in smart cities combines smart technologies, smart wearable devices and multi-agent sensors to support smart e-healthcare applications which can build a smart healthcare city. Nowadays, numerous initiatives have been taken to encourage the continuous monitoring of people’s health condition through smart wearable devices like fitness bands and health monitoring apps in smart phones. These devices aim not only at monitoring health status continuously, but also at providing needed solutions at the right time. The smart wearable devices help to remotely assess individual health status or fitness regime without any professional help. To name a few applications, these devices can be used to help check blood and glucose level, body temperature, heartbeat, cardiovascular problems, vision quality and chronic ailments. Smart-city healthcare technology interacts with smart devices, collects data produced by these devices, and finally transfers these to doctors, researchers and the healthcare experts for better personalized diagnosis and solutions. In order to collect, analyze, process and suggest the best diagnoses, we need to integrate a system which is capable of performing different operation wisely and effectively.

MAS is a paradigm of great importance because a system capable of learning and changing its way of acting dynamically provides a great potential to face many problems the behavior of whose agents in the environment we do not know. This adds more levels of difficulty in tasks of consensus and coordination between agents, as they may be learning at all times and changing their behavior. A MAS is a distributed autonomous system consisting of multiple agents, who are autonomous in computing with good knowledge and solving capability. All these agents work collectively to provide effective healthcare to the citizens living in the city. Thus, a MAS approach can be considered as an effective approach to design and implement for the following reasons:

i. Provide an opportunity to divide the problem into subproblems solved by agents present in the MAS working as a team for defining and integrating information from different healthcare units to process the information efficiently;
ii. Propose best medical diagnostics to the patient based on the information collected from the patient;
iii. Provide coordination between different units and between the actors involved in the treatment process and tries to optimize the exchange of data between the units.

The advantages of MAS based e-healthcare systems include:

- Efficiency: Increase efficiency in patient healthcare makes decrease in costs.
- Increase in quality of care: e-health may improve the quality of patient healthcare by directing patient health information to the best quality diagnosis providers.
- Encouragement of a new association between the patient and health proficient, towards a true partnership, where decisions are made in a shared manner.
- Education of physicians through online sources (continuing medical education) and consumers (health education, tailored preventive information for consumers).
- e-health permits patients to easily obtain health services online from global providers. These services can range from simple advice to more complex interventions or products such as pharmaceuticals.

As many numbers of zones are connected with each other through city healthcare management, it is highly impossible to provide efficient smart e-healthcare system with single software. In order to facilitate better smart healthcare system, the functionality should be divided into sub operations so that it can be autonomously monitored by a single software or hardware unit. Fortunately, MAS is an autonomous unit responsible for doing particular operations. Hence, the integration of MAS in smart city e-healthcare system will improve the efficiency. In recent days—taking the healthcare of citizens into the consideration—governments, along with public private partnerships are investing significantly in these projects. In year 2015, Dubai (UAE) established a unified national health database [4], which connect all hospitals and clinics for creating effective database concerning patient’s medical history, ailments, surgeries and tests conducted. The aim is to save patients and help doctors perform diagnoses in an effective manner. In the South Pacific region, Australia initiated telemedicine and telehealth services [5], this improving both public and private hospitals.

As the data coming from the wearable devices communicate via wireless communication, the drawback for smart city healthcare is security and privacy issues. The main concern should be in terms of medical ID cards, which contains the personal details of the patient. In addition, hospitals must ensure security in encryption while issuing data collected for further processing for the benefit of the mankind. Hence, the security aspects of e-healthcare MASs are critical for providing security, privacy of healthcare data and safety of citizens in smart cities is the need of the hour. In this line of research, we propose a novel mechanism for securing the communication in distributed e-healthcare sensor systems with quantum principles, advancing the up-growing field of quantum-based security. In this way, e-healthcare MASs will be secure—even when quantum computing is able to be used to hack sensor systems.

## 2. Related Work

Different methodologies have been proposed earlier in for maintaining the privacy and security of the electronic health records (EHR) [6,7]. However, these methods need more security in order to distribute the health-related data. The e-healthcare systems are real time and have patient information which is in digital format. These are maintained by licensed persons. These data sets where formed by acquiring various data from different patients. In these EHRs, the authorized persons can be the patients or the doctors. The data present in the servers can be available in local or cloud, which stores and analyses the stored health data. The components which are present in the networks can be the inter connector between the patients and the medical staff for enhancing the broadcasting and distribution of data. However, there are many benefits to these systems: more threats are present in terms of security and privacy for the data used therein. These security threats are inherent to the system design. These threats can be classified into various categories such as data collection level [8,9,10], transmission level [11,12,13,14] and storage level [15,16], which are described more clearly in Section 3. Due to these threats in security and privacy of the EHR data, some users are not ready to use these applications. Hence, it is necessary to ensure that users should be ready to use the system without any hesitation. Therefore, it is important to propose a system for maintaining the security in the EHR data.

Quantum key distribution (QKD), started with a protocol BB84 by Bennett et al. [17] in 1984, addressed how to share an arbitrary key between 2 parties via single qubits using the quantum channel. The security of QKD uses arbitrary measurements of the qubits in one of two non-orthogonal bases, complementary and the fact that quantum mechanics rule out an eavesdropper from getting hold of information on the state of an unfamiliar qubit without upsetting it. In this way, any ensuing estimation of a complementary apparent on the same qubit becomes arbitrary. Moreover, entanglement and the superposition characteristics allow investigators to build up the quantum algorithm (QA) used to break the renowned RSA cryptosystem by quantum parallel computing. A QA can be a powerful weapon to intimidate conventional cryptography. It allows investigators to build up quantum cryptography, which offer security using on physical laws rather than computational complexity, to shield in opposition to attacks from quantum computers. Moreover, further appealing applications conflicting the history are improved, such as quantum dense teleportation and coding. As far as this, three appealing branches of quantum cryptography are QKD [18,19,20,21], quantum secret sharing (QSS) [22,23,24,25] and quantum secure direct communication (QSDC) [26,27,28,29].

In 1991, Ekert developed an enterprise resource planning (ERP) based algorithms as a first QKD convention named E91 [30]. In the following year, an improvement was made by Bennett C; (H) and proposed an algorithm [31], which utilizes non symmetrical bases and two qubit states. Afterwards, in 2004, research was diverted onto key rate, key usage and storage space in bidirectional QKD. In 2004, Nguyen, proposed an algorithm [32] which permits two agents to swap over their secret message in one transmission, as a bidirectional QSDC (BQSDC) protocol or quantum dialog bidirectional. Gao et al. [33] in 2005, enhanced the protocol that adds a control head to assist the recipient to decrypt cipher message, which is exclusive of earlier knowledge about the message. Moreover, Jin et al. invented a multiparty QSDC (MQSDC) protocol [34], which permits agents to interchange their secret contributions, concurrently. Furthermore, Deng et al., [35]; Zhang et al., [36]; Chou et al. [37] and Hwang et al. [38] all invented competent multiparty QSS (MQSS) protocols during 2005 to 2012. Subsequently, Jia et al. [39]; Liao et al. [40]; Hsu et al. [41] and Liu et al. [42] as well introduced the proposal of dynamic MQSDC (DMQSDC) for the period of the period of 2012 to 2016.

Many quantum cryptographic algorithms [43,44,45,46,47,48,49,50] were proposed by diversified authors, which were widely used in many modern applications. Still, the progress of quantum key Agreement (QKA) is the significant subtopic in QKD. In QKD, one-member fix on the key and then distributes it to the other members, where as in QKA, more than one participant will be involved in key derivation. The QKA aiming to collect the pieces from all or selected participants to create a secret key.

The idea of multiparty QKA (MQKA) was first presented in 2012 when Shi et al. [51] proposed the foremost MCQAP dependent on Bell estimation and Bell states. After this, Liu et al. [52] brought up the drawbacks in this protocol, and afterward proposed one more MCQAP utilizing single particles. Since this point, many more MQKA protocols have been proposed. In 2013, Sun et al. [53] made the endeavor to improve the productivity of Liu et al.’s. MCQAP and propose a MCQAP in traveling mode. Unevenly, this protocol has additionally been shown to be unfair [54]. In 2014, an appropriated mode MCQAP is proposed with GHZ states by Xu et al. [55]. Around the same time, two traveling mode MQKA protocols were given cluster states and six-qubit states, separately by Sun et al. [56,57,58]. In the interim, however, these traveling type of MCQAP agreements proposed in these are out of line with collusion attack, i.e., a nontrivial subset of the group members can conspire to find the final shared key devoid of being noticed by others. In 2016, Huang et al. introduced a traveling mode MCQAP with single photons and unitary tasks [59]. Recently, Cao et al. also introduced a traveling mode MCQAP dependent on quantum search algorithms [60].

In turn—to attain the key generation setting and the generalization of two-party to MQKA—these MQKA protocols utilize the unicast communication method, swapping information one-for-one basis. Like this, the resource utilization will increase rapidly with the increase of members. In 2016, Zeng et al. [61] introduced a proficient MCQAP that relies on MQSDC utilizing ‘broadcast’ transmission, which implies that all agents can trade their mystery message, greatly improving effectiveness—yet additionally conserving time and quantum asset. In this work, we propose a MQKAP which can oppose both outer and inner attacks. In contrast, the proposed method utilizing the multicast transmission protocol is more viable than other current MQKAP. The proposed technique is based on the idea of generalization of two-party QKA to MDQKAP/ DQGKA. We expect the results of the proposed work will be useful for advanced research on fair MQKAPs. In this paper, we effectively use the proposed MDQKAP/DQGKA for secure communication in e-healthcare multi-agent system in smart cities.

### Contributions

The main contributions of this work are indicated below:

- Propose a three-layered architecture for zonal healthcare systems;
- Propose a schematic arrangement of multi-agent system in zonal healthcare system which facilitates improvement in quality of healthcare;
- Propose a quantum group key agreement (QGKA) suitable for secure communication among multiple agents to achieve security in sharing the patient information;
- To prove the performance of proposed protocols is efficient in terms of Qubit efficiency (QE), unitary operation (UO), unitary operation efficiency (UOE), key consistency check (KCC), security against participants attack (SAP).

The remainder of this article is organized as follows. Section 3 presents the background of the protocol, two party QKA protocol with single photons. Section 4 presents the proposed methodology. Section 5 discusses the experimentation environment and the results. Section 6 presents the comparative analysis of the proposed work with other existing methods, considering fairness and security. The last section mentions a concise conclusion and future work.

## 3. Background Protocol for Quantum-Based Security in e-Healthcare Multi-Agent Systems

### 3.1. Notations

Here we specify all notations used in this paper. Table 1 indicates the list of abbreviations used in this article.

### 3.2. Outline of Quantum Two-Party Key Generation

Key generation plays a vital role in all cryptosystems, which is used for encrypting as well as decryption of messages. To understand the basics of key agreement for background quantum concepts, one can refer a quantum Diffie–Hellman protocol. Although this work is focused on its particular application of e-healthcare MASs, this generation mechanism is general to most kind of systems. 

**Figure 1:**
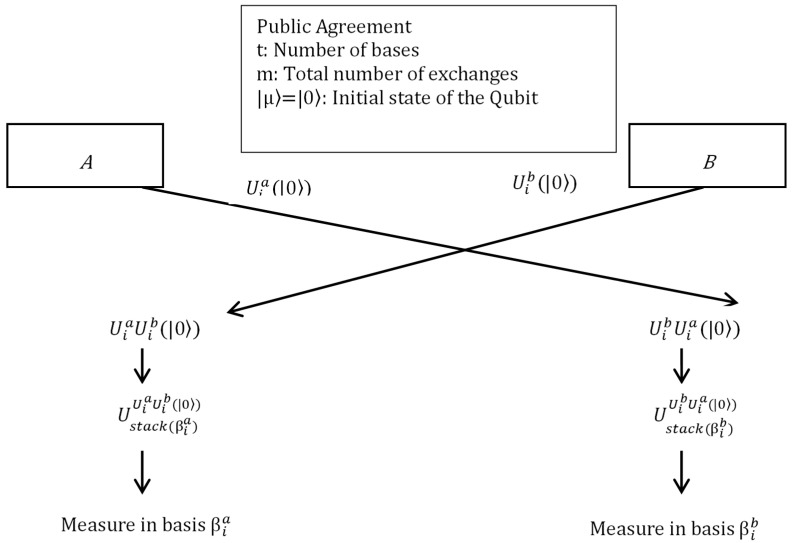
Outline of quantum two-party key-generation protocol.

In this direction, first we present the outline of the established two-party key agreement using quantum operations as depicted in Figure 1. At the end of the process, both A and B parties are left with a common shared key.

### 3.3. Two-Party Quantum Key-Generation

**Figure 2:**
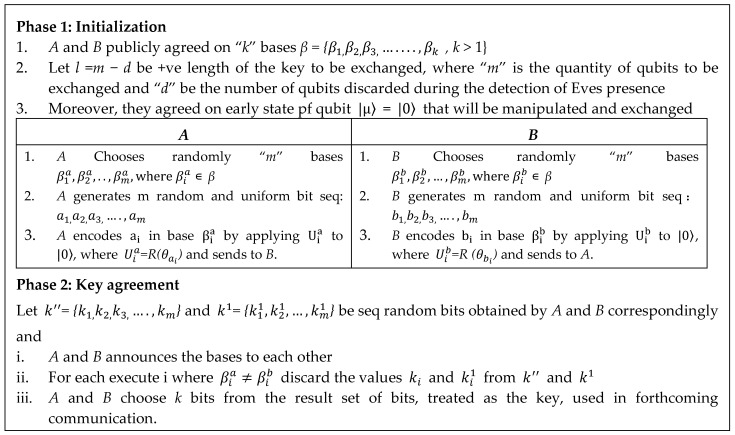
Two-party quantum key agreement algorithm.

Figure 2 shows the complete analysis of two-party quantum *k* bit (*β =* {$\beta_{1,}\beta_{2,}\beta_{3,}\ldots ,\beta_{k}$*, k* > 1}) key agreement. Initially, both users involved in communication publicly decide *k* bases; also the *m* value (number of qubits used in key agreement). Each user individually selects *m* random bases $\beta_{1}^{a},\beta_{2}^{a},\ldots ,\beta_{m}^{a},\;{\mathrm{where}\;\beta }_{i}^{a}∊$ *β* and $\beta_{1}^{b},\beta_{2}^{b},\;\ldots ,\beta_{m}^{b},{\;\mathrm{where}\;\beta }_{i}^{b}∊$ *β*, also, *m* random bits $a_{1,}a_{2,}a_{3,},\ldots ,a_{m}$ and $b_{1,}b_{2,}b_{3,}\ldots ,b_{m}$, then calculates $U_{i}^{a}$ to |0〉, finally sends to other participants.

After receiving the keys from the other user, the key finalization process is initiated by discussing publicly about the used basis. The key bit is accepted in final key when the predicted key bit is matched with the bases used. Otherwise, it is discarded. This operation continues until all the m bits are processed. The bits satisfying the similarity check are be the final key used for encryption and decryption.

## 4. Proposed MAS Based e-Healthcare System Architecture

In this section, first we propose a zone level three-layered system architecture. Next, we present the MAS arrangement to facilitate effective processing in the zonal-level architecture. To address secure communication in the proposed architecture, we present a quantum-based group-key agreement. Finally, we integrate the above to establish MAS based e-healthcare system in a city named as smart healthcare city.

### 4.1. Zonal Level Healthcare System Architecture

**Figure 3:**
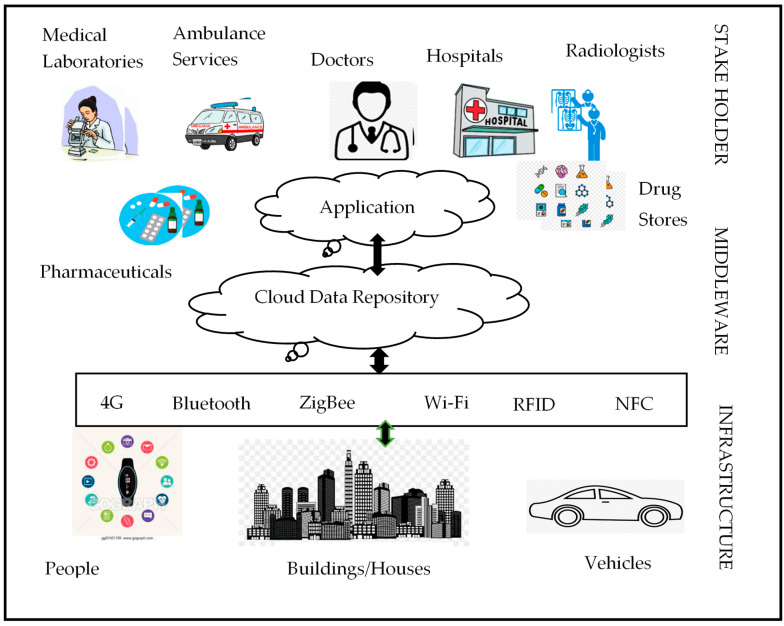
Zone level healthcare system architecture.

In this subsection, we present a three-layered architecture of the zonal-level healthcare system as depicted in Figure 3. This is named as the infrastructure layer, middleware layer and the stakeholder layer.

**A.**  **Infrastructure Layer**: This layer is the patient–data–producer layer. It collects the health data from wearable devices, WBANs, houses/buildings and the sensors attached to the vehicles. All the collected data will be transferred to the middleware layer through communication protocols like 4 G, Bluetooth, Zigbee, etc.
**B.**  **Middleware Layer**: This layer’s responsibilities include data collection from the infrastructure layer, data processing, data storage on local data storage area and sharing of the information with the concerned stakeholder for further processing of the patient data. It is also responsible to transfer the data to the central healthcare management system.
**C.**  **Stakeholder Layer**: This layer consists of the all the healthcare experts who can able to work on patient health records. The stakeholders in this layer includes doctors, laboratories, pharmaceuticals, ambulance services, medical stores, radiologists, etc. As the patient data are collected from the middle layer, it will be dispatched to relevant stake holder in order to provide qualitative diagnosis. It also responsible to share the patient health records with the research laboratories when the doctors failed to trace the disease for analysis, in the continuation, the drugs can be manufactured by the pharmaceutical’s companies.

### 4.2. Smart Healthcare Architecture

**Figure 4:**
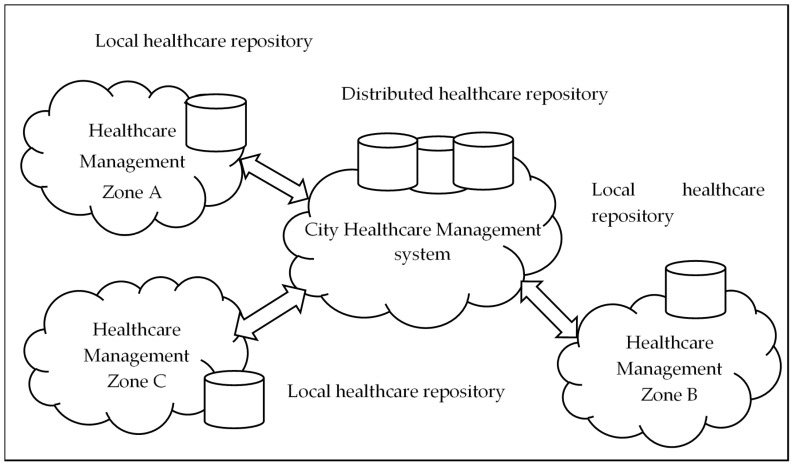
Smart city healthcare architecture.

In this subsection the smart city healthcare architecture is depicted in Figure 4, this arrangement is connecting the healthcare system. Traditionally, smart city is divided in zones for better administration and each zone has its own healthcare system. Keeping in view, fast and effective data processing and technical challenges, it is assumed that smarter city objective will be achieved in an incremental manner. We used the concept of zone level service; arrangement supports step-by-step movement towards a smart city. Each zone has its own autonomous healthcare system that comprises a local data collection center, communication infrastructure and local stake holder.

In Figure 4 we can observe that, a city healthcare management system, is connecting the healthcare system of three zones termed as zone A, zone B and zone C. The responsibilities of the city healthcare management include, patient health information collection from different zones, storing of collected patient health information in master repository and sharing of health record of patient in one zone to stake holders in other zones to provide better diagnosis.

**Figure 5:**
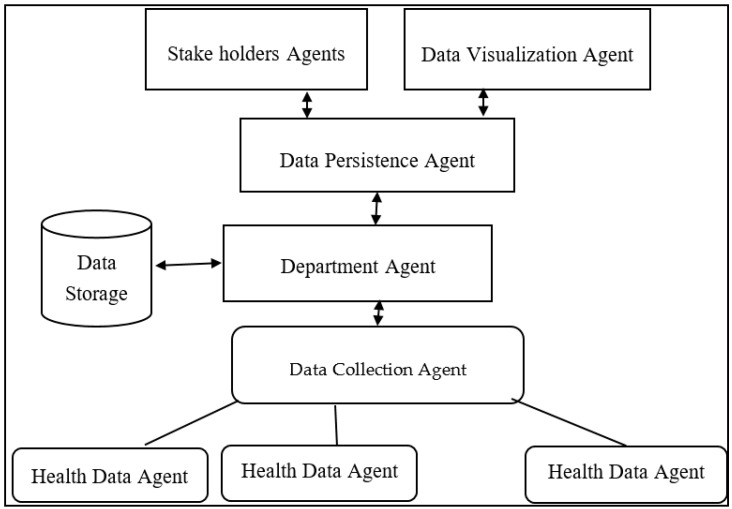
Multiagent architecture of smart city healthcare system in a zone.

As many numbers of zones are connected with each other through city healthcare management, it is highly impossible for one software to provide efficient smart healthcare system. In order to facilitate better smart healthcare system, the functionality should be divided into sub operations and then autonomously monitored by a single software or hardware unit. Fortunately, MAS is an autonomous unit responsible for doing particular operation. Hence, adapting MAS in healthcare provides quality service to the patient effectively. Figure 5 shows the zone-wise MAS, consisting of the following agents:

1. Health data agent: These are nothing, but the primary patient health recording devices, continuously monitor the health parameters of the patient, forward the health information to the data-collection agent. In general, the health data agents can be wearable devices, WBAN networks, etc. In addition to monitoring of citizen health, it is also responsible to transfer the collected information to the nearby data-collection agent;
2. Data-collection agent: This is the unit placed at the region wise, it is responsible to collect the data from all the devices (citizen wearable devices, building/houses, vehicles, etc.), and then transfer it to the department agent;
3. Department agent: The department agent is the software placed in the zone head office, responsible to collect patient data from all the regions in connection with it, this agent usually presents in the middle layer. The responsibilities of the department include, data processing using any of the machine learning algorithms, and then place it onto the data storage unit;
4. Data persistence agent: The data persistence agent is responsible to extract a piece of required patient data present on the database storage unit and then forward it to data visualization unit as well as to the stake holder agent;
5. Stake holder’s agent: The stakeholders’ agent is usually present in top layer, is responsible to deliver the extracted patient information to the required stakeholders includes, booking an appoint to doctor, sending alert to ambulance, etc.

Hence, smart city e-healthcare system is the arrangement, which integrated different zones through the MAS. As we already aware of that, communication between multiple agents raises security and privacy challenges. In order to provide, security to the patient information, which is passing through multiple agents at multiple levels need to be secured. In this paper, we are proposing a quantum group key agreement protocol to secure the patient health information. The key can be used in encryption and decryption algorithms to secure the information. The provision of security algorithm based on fundamental laws of physics, the scope of unhackability and easiness in usages made us to prefer quantum key cryptography in key agreement between the agents.

### 4.3. Quantum Group Key Agreement Protocol

Let $M_{1,}M_{2,}M_{3,}\ldots ,M_{n}$ be the members of the group.

**Public agreement:** All the group members $M_{i,}\;1\leq i\leq n$ agree on the set of *t* bases $\beta_{1},\beta_{2},\ldots ,\beta_{t},$ for *t* > 1 to use and the quantity of qubits to exchange. The value of *m* is based on the required key length and discarded quantity of qubits during the detection of presence and errors.

**Step 1:** In this step the group controller forms two parties with remaining members and generates QDH two-party keys, respectively, as follows:

The GC $M_{1,}$ generates two party keys with $M_{i,}$ 2 ≤ *i* ≤ *n*.

$$QDH_{M_{1}M_{i}}\left(t\right)\to k_{1,i,}2\leq i\leq n$$

In this step of proposed protocol in the process of generating two-party keys, entanglement allows legitimate parties to detect eavesdroppers by virtue of the fact that if the sender and recipient each have a photon the two of which are related by quantum mechanical entanglement -interception or measurement by an adversary will change the two photon system in a way that the legitimate parties can readily detect.

**Step 2:**$\mathrm{The}\;\mathrm{GC},\;M_{1}\mathrm{computes}\;\mathrm{the}\;\mathrm{partial}\;\mathrm{key}\;\mathrm{component}\;“{⊕}_{i=2,i\neq j}^{n}k_{1,i}$” for each member $M_{i}$ and encrypt them with the respective to members shared key and send them to the respective members as follows:

$$M_{1}\overset{E_{k_{1,j}}[{⊕}_{i=2,i\neq j}^{n}k_{1,i}]}{\to }\;M_{j},\;2\leq j\leq n$$

**Step 3:** After receiving the message from $M_{1}$ each group member $M_{j},$ decrypts the partial key component with respective key and XORed it with their own shared key to computes the group key as follows.

$$Group\;Key=QGDH_{M_{1}M_{2\ldots }M_{n}}(t)={⊕}_{i=2}^{n}k_{1,i}$$

**Step 4:** Finally, as the GC knows all the two-party keys, he can easily compute the group key by joining all the two-party keys with EX–OR as $Group\;Key\;{⊕}_{i=2}^{n}k_{1,i}$.

### 4.4. Dynamic Quantum Group-Key Agreement Protocol

The GKA protocol, QGKA, is primarily suitable for static groups, in which the group members are fixed. However, in spite of the existing group members, there are the scenarios where, we need to add a new member (or) delete an existing group member from the initial group. To address this dynamic connectivity of nodes in group, join and leave protocols are added to QGKA technique is termed as DQGKA.

In this subsection we extend the quantum group key agreement protocol by proposing member join protocol and member leave protocol

#### 4.4.1. Member Join Protocol

This protocol will uphold the secrecy of the earlier group key even after join of new members in the group.

i. Once per fresh member *M_n+1_* need to add into group, it informs the GC and produce *QDH* key $k_{1,n+1}$ with GC by taking the advantage of *QDH*.
ii. The GC produces *r_n_*_+1_ a random quantum string and broadcasts *k*_1,*n*+1_⊕*r_n_*_+1_ to group members *M_i_* present before. Upon getting, new GK is calculated as
  $$NJKA=PGK⊕k_{1,n+1}⊕r_{n+1}={⊕}_{i=2}^{n+1}k_{1,i}⊕r_{n+1}$$
iii. The GC sends out *PGK* ⊕*r_n_*_+1_ *to M_n_*_+1_. Now *M_n_*_+1_ compute the fresh key as
  $$NJKA=PGK⊕r_{n+1}⊕k_{1,n+1}={⊕}_{i=2}^{n+1}k_{1,i}⊕r_{n+1}$$

#### 4.4.2. Member Leave Protocol

This protocol secures the new group key derived by current group members from the members leaving the group along with the outsider.

This protocol will secure the new group key derived by current group members from the members leaving the group along with the outsider.

(i) Once *M_j_* desires to leave from group, it informs the group controller.
(ii) The GC produces $r_{j}$ a random quantum string and sends out $k_{1,j}⊕r_{j}$ by enciphering with $k_{1,i}$ to the respective group member *M_j_*, *i* ≠ *j*, i.e., excluding members left from group.
  In other words, $M_{1}\overset{E_{k_{1,i}}\left[k_{1,j}\;⊕r_{j}\right]}{\to }M_{j}$, for 1 ≤ *i* ≤ *n*, *i* ≠ *j*.
(iii) On getting, group member *M_i_* decipher the received message with $k_{1,j}$ and calculate the fresh key as under:
  $$NLKA=PGK⊕k_{1,j}⊕r_{j}={⊕}_{i=2,i\neq j}^{n}k_{1,i}⊕r_{j}$$
(iv) In addition, *M_L_* calculates new key as:
  $$NLKA=PGK⊕k_{1,j}⊕r_{j}={⊕}_{i=2,i\neq j}^{n}k_{1,i}⊕r_{j}$$

Table 2 shows the computation and communication cost of the DQGKA for different operations like group initialization, member join and member leave operations.

## 5. Security Analysis of the Proposed Quantum Based Approach for Securing MAS Based E-Healthcare System

In this section, first we discussed the performance and security of QDH protocol and then show that the proposed protocol DQGKA presented in Section 4.3 and Section 4.4 all have the same inherent security features, such as key secrecy, forward secrecy, backward secrecy. Further we present protection from some of the important attacks such as internal eavesdropping, intercept and resend attack with fairness analysis.

### 5.1. The Performance and Security of QDH Protocol

Alice and Bob *m* > 1. Let *s*, 0 ≤ *s* ≤ *m*, be the number of usable qubits obtained from these exchanges, i.e., the number of qubits for which Alice and Bob selected the same bases. Let σ be the fraction of s compared to detect Eve. Therefore, number of comparisons performed by Alice and Bob to detect Eve is *k* = *s* × *σ*. The number of bits in the secret shared key is *l* = *s* − *k*. The number of usable qubits obtained in QDH (t) depends on two factors:

- the number of bases used, *t*;
- the number of exchanges performed, *m*.

Since Alice and Bob independently, randomly and uniformly probability that they will choose the same exact basis, for any given exchange, is 1/*t*. Then the number of usable qubits obtained from m exchanges is *s =* $\frac{m}{t}$.

The efficiency of the protocol, ρ, is then defined as the ratio of the number of usable qubits to the number of exchanges performed in the protocol. This result in, ρ = $\frac{s-\left(\sigma \;\cdot \;s\right)}{m}$ = $\;\frac{\;1-\sigma }{t}$.

The security of QDH (t) depends on Alice and Bob performing a sufficient number of exchanges so that Eve can be detected with high probability. The sum 1 − *σ* of the usable qubits result in bits that constitute the shared secret key.

The probability of detecting Eve for a given number of exchanges *m* and number of bases *t* is computed as follows:

In the QDH (t) protocol, *k* of the *s* usable qubits are used to detect Eve and the remaining *s* − *k* qubits constitute the key whenever Eve is not detected. The probability of Eve not being detected in these *k* exchanges.

In general, in a protocol QDH (t), *t* > 1 available bases, the probability of Eve measuring at least one of the qubits with a basis other than that chosen by Alice and Bob *P(A) =* $\frac{t^{2}+1}{t^{2}}$. The probability of the measured qubits producing different values *P(B/A) =* $\frac{1}{2}$.

Note that *P(B/A)* is computed assuming that all of the four-qubit pair value 00, 01, 10 and11 are equally likely. This is the case whenever the base angle chosen by Eve differs from that of Alice and Bob in a way that the qubit measured by Eve collapse to 0 and 1 with roughly equal probabilities.

Therefore, the probability of detecting Eve in QDH (t) is, *P_d_* = 1 − ${\left(\frac{t^{2}+1}{2t^{2}}\right)}^{k}$.

- With the increase in number of bases, *t*, is increased; we see that probability of Eve going undetected tends to one-half (for each exchange) and the probability of detection in *k* comparisons becomes:
  $$P_{d}=1-{\left(\frac{1}{2}\right)}^{k}\;\mathrm{as}\;t\to \infty .$$
- Our results showed that with the increase in number of comparisons *k*, probability of detecting Eve in QDH (t) increases.
- Eve can be detected with a probability *P_d_* = 0.5, even when *k* = 1.

### 5.2. Security of Proposed Protocol DQGKA

**Theorem** **1.** *The group key derived using quantum group key agreement protocol is indistinguishable in polynomial time from random numbers.*

**Proof.**  Each of the two-party shared keys generated in the Step 1 of quantum group key agreement protocol is secure, because it uses a QDH protocol. That is, all the two-party shared keys exchanged in Step 1 are indistinguishable from random numbers in polynomial time.

The GC $M_{1,}$ generates two party keys with $M_{i}$, 2 ≤ *i* ≤ *n*.

$$QDH_{M_{1,}M_{i}}\left(t\right)\to k_{1,i,}2\leq i\leq n$$

In this step of proposed protocol in the process of generating two-party keys, entanglement allows legitimate parties to detect eavesdroppers by virtue of the fact that if the sender and recipient each have a photon the two of which are related by quantum mechanical entanglement -interception or measurement by an adversary will change the two photon system in a way that the legitimate parties can readily detect. Hence, that man in the middle attack can be easily detected.

In Step 2 $\mathrm{The}\;GC,\;M_{1}\;\mathrm{computes}\;\mathrm{the}\;\mathrm{partial}\;\mathrm{key}\;\mathrm{component}\;“{⊕}_{i=2,i\neq j}^{n}k_{1,i}$” for each member $M_{i}$, respectively and send them to the respective members.

$$M_{1}\overset{E_{k_{1,j}}[{⊕}_{i=2,i\neq j}^{n}k_{1,i}]}{\to }M_{j},\;2\leq j\leq n$$

Note that these partial key component is also secured as it is obtained by XORed the secured shared keys generated in Step 1. Further, the GC sends these partial key components to the respective group members securely through established encrypted link between them. After receiving, the respective member decrypts their partial key component and computes the group key securely by XORing with own shared key as follows:

$$\mathrm{group}\;\mathrm{key}=QGDH_{M_{1}M_{2\ldots .}M_{n}}(t)={⊕}_{i=2}^{n}k_{1,i}$$

Since the group key ${⊕}_{i=2}^{n}k_{1,i}$ is indistinguishable from random numbers in polynomial time, and thus secured. □

**Theorem** **2.** *The join protocol of DQGKA satisfies the properties of backward security.*

**Proof.**  This protocol will uphold the secrecy of the earlier group key even after join of new members in the group as follows:

(i) Once per fresh member M*_n_*_+1_ need to add into group, it informs the GC and produce QDH key *k*_1,*n*+1_ with GC by taking the advantage of *QDH*.
(ii) The GC produces *r_n_*_+1_ a random quantum string and broadcasts *k*_1,*n*+1_⊕*r*_n+1_ to group members Mi present before. Upon getting, new GK is calculated as *NJKA = PGK*⊕*k*_1,*n*+1_⊕*r_n_*_+1_ = ${⊕}_{i=2}^{n+1}k_{1,i}$⊕r_*n*+1_.
(iii) The GC sends out *PGK*⊕*r_n_*_+1_ to *M_n_*_+1_. Now *M_n_*_+1_ compute the fresh key as *NJKA = PGK*⊕*r_n_*_+1_⊕*k*_1,*n*+1_ = ${⊕}_{i=2}^{n+1}k_{1,i}$⊕r_*n*+1_.

As in step 3 the GC sends out *PGK*⊕*r_n_*_+1_ to *M_n_*_+1_. Since *M_n_*_+1_ does not know *r_n+_*_1_ it cannot find PGK and hence, backward secrecy is attained of join protocol *DQGKA*. □

**Theorem** **3.** *The leave protocol of DQGKA satisfies the properties of the forward security.*

**Proof.**  This protocol will secure the new group key derived by current group members from the members leaving the group along with the outsider.

(i) Once *M_j_* desires to leave from group, it informs the group controller.
(ii) The GC produces $r_{j}$ a random quantum string and sends out $k_{1,j}⊕r_{j}$ by enciphering with $k_{1,i}$ to the respective group member *M_i_*, *i* ≠ *j*, (i.e.,) excluding members left from group.
  In other words, $M_{1}\overset{E_{k_{1,i}}\left[k_{1,j}⊕r_{j}\right]}{\to }M_{i}$, for 1 ≤ *i* ≤ *n*, *i* ≠ *j*.
(iii) On getting, group member $M_{i}$ decipher the received message with $k_{1,j}$ and calculate the fresh key as under:
  $$NLKA=PGK⊕k_{1,j}⊕r_{j}={⊕}_{i=2,i\neq j}^{n}k_{1,i}⊕r_{j}$$
(iv) In addition, *M_L_* calculates new key as:
  $$NLKA=PGK⊕k_{1,j}⊕r_{j}={⊕}_{i=2,i\neq j}^{n}k_{1,i}⊕r_{j}$$

To exclude the shared key component $k_{1,j}$ of leaving member $M_{j}$ from *PGK*, we use *PGK*⊕$k_{1,j}$. To make the updated key secure from $M_{j}$, the GC includes another random $r_{j}$. Hence, we have the principal security prerequisite of member exiting holds with respect to previous members of the group and outsiders. □

### 5.3. Attacks on Proposed Protocol DQGKA

**Protection from Internal Eavesdropping:** In fact, inside members of the group have greater capacity to attack than the outsiders. The untrustworthy nature of inner members, who could get the advantage from replacing the sequence of messages with the fascinated sequence, in turn to stay away from these, commence an internal attack in opposition to the group through his acquired the assets. As the proposed quantum group key agreement is contributory in nature provided the GC should be trustworthy. Hence, that the internal members of the group cannot influence the group key. Thus, the proposed protocol is protected from the eavesdropping attack of internal members.

**Intercept and resend attack:** As assault, Eavesdropper Eve (E) attempts to quantify the quantum states originating from A and afterward sends the changed states to B. As E, has no information about basis of state chosen by A, he/she can just estimate which basis to measure in, similarly B does. In the event that E picks effectively, she/he can measure the right photon polarization state as it is sent by A and reacts in right state to B. If E picks wrongly, then state estimation is random, and the state conveyed to B is not as sent by A. Table 3 below shows an instance of this attack.

The likelihood E picks the wrong basis is 50% (supposing A picks arbitrarily), and if B measures this intercepted photon in the basis *A* gets an arbitrary result, i.e., a wrong outcome with likelihood of 50%. The likelihood of an intercepted photon creates an error in the key string is then 50% × 50% = 25%. If *A* and *B* openly contrast their key bits (thus leaving them as key bits, as they are no longer secret) the likelihood they discover variance and recognize the existence of E is $P_{e}=1-{\left(\frac{3}{4}\right)}^{n}$.

Hence, to notice an eavesdropper with probability $P_{e}=0.999$. *A* and *B* require to compare n = 72 key bits. In the proposed protocol entanglement allows legitimate parties to detect eavesdroppers by virtue of the fact that if the sender and recipient each have a photon the two of which are related by quantum mechanical entanglement -interception or measurement by an adversary will change the two photon system in a way that the legitimate parties can readily detect.

**Fairness Analysis:** It is recognized fact that, in a group quantum cryptographic protocol, there is a possibility of attack from fraudulent members either external or internal. These members are having more scope to attack the protocol. Initially, he/she is able to change the legal photon sequence, next, bring in errors into the information. Further they may team up with some more unfair members in implementing the protocol. As the proposed protocol is using the concept of GC, the secured communication is happening between GC an individual participant in the group, even though all the participants can able to compute the shared key. For communication between two participants, it is proved that the proposed mechanism is secured against eavesdropping in previous section. Hence, proposed protocol is secured against participant attack.

## 6. Results and Discussion in the Context of Multi-Agent-Based e-Healthcare System in Smart Cities

In order to test the current approach, we have focused on common interactions in multi-agent system in smart cities, like in the context of doctor’s appointment data set smart, this application belong to the context of smart cities, as the proposed approach is aimed at securing multi-agent system for e-healthcare in smart cities.

IBM developed a composer, suitable for quantum computing called as Qiskit, which can be used for real time experiments like quantum simulation, quantum algorithms development, testing of theoretical tasks, quantum cryptography and error correction. Qiskit comprises of four central components: Terra (the code establishment, for forming quantum programs using circuits and pulses), Aqua (creating algorithms and applications), Ignis (removing noise and correcting errors) and Aer (quickening improvement by means of emulators, simulator and debuggers). In our experimentation, Aer, the ‘air’ component, pervades all Qiskit components. Hence, as to accelerate the improvement of quantum PCs better emulators, simulators and debuggers are required. Aer will assist us with understanding the cutoff points of traditional processors by showing to what degree they can copy quantum calculation. Moreover, we can use Aer to validate that present and near-future functions of quantum computers properly. This should be possible by extending the cutoff points of simulation and by recreating the impacts of practical noise on the calculation. Algorithms implementation has done using Aer elements in Qiskit. The generated quantum key is used to encrypt the dataset with the following information. In the experimentation, a doctor appointment dataset (110,356 doctor appoint records) is taken from the Kaggle [62] with 14 columns named as PatientID, AppointmentID, Gender, Scheduled Day, Appointment Day, Age, Neighborhood, Scholarship, Hypertension, Diabetes, Alcoholism, Handicap, SMS_received, No-show.

The proposed algorithm initially sets up a group with m users (healthcare agents or stake holders) among them the first user will act as the group controller (GC) aiming to share a common key among the group users. In the experimentation group is created for m = 5 users, user-1 becomes GC and other four are group members. Once the group users are identified, they decides the key length(n), in the experimentation the max key length used is 23 bits, generates possible numbers in range 0 to 2^23^ − 1.After the finalization of the bit length, each user creates their own register with the bit length (n) in order to store n bits using the function quantum Register(). Each individual user will start sharing their contribution with GC, Table 4 shows the quantum key-sharing process between individual group member and the GC for a key length 10. Then, GC initiates a process to calculate the key to individual user from the received keys. For user-2 GC uses the keys received from other users except user-2, performs the XOR operation on these keys. Similarly, he follows the same procedure in computation of all other group users and communicates securely to the users, key calculation formula for user-i is given by $PK_{gci}=⊕\left(K_{j}\right)$ for all *j* ∈ {1,*n* − 1} and *j* ≠ *i*, where $PK_{gci}$ is the partial key computed by GC for i-th member.

Table 5 shows the partial key component calculated by GC for these four users in the group. Afterwards, GC calculates the common group key by performing the XOR operation on all the keys of group users along with his key, it is calculated as $PK_{gci}$.

Table 6 shows the final group key calculated by GC, all other group members. Group key is computed by XOR the received partial component from GC with his own key contribution, which is given by $Key_{i}=PKey_{GCi}⊕K_{i}$, here $Key_{i}$ established by each member will be the same, which we can use as a group key.

Algorithm development has done using the Qiskit built in functions, its description is shown in Table 7, each user in the group needs to create a register based the qubit length using QuantumRegister() function, then after a random number is generated using np.random.randin(),returns any number in the range 0 to 2*^n^*^−1^, np.binary_repr() function is used to convert it into binary form in order to store on register.

**Figure 6:**
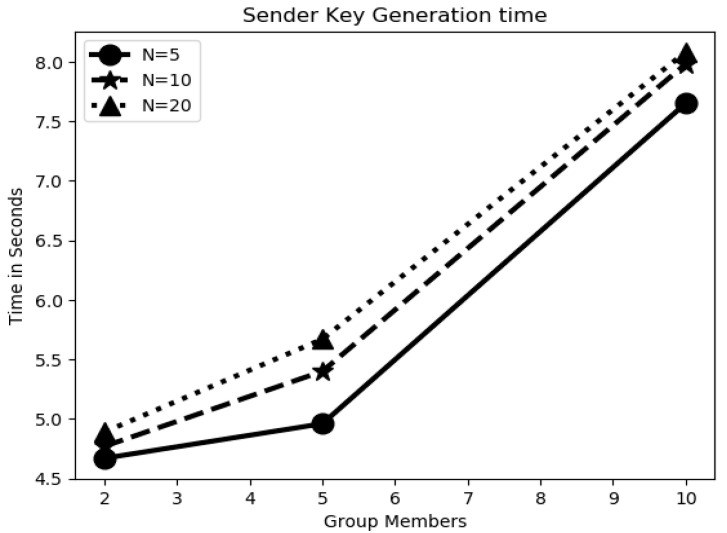
Key-generation time among multi-agents/stake holders.

**Figure 7:**
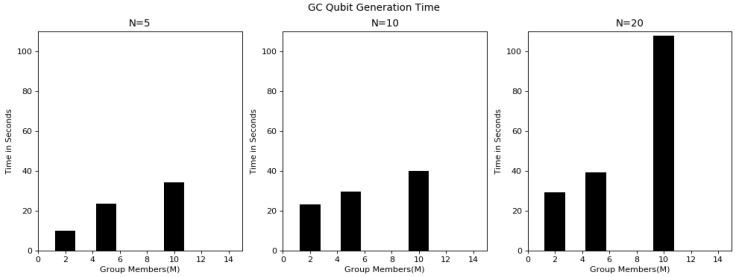
Time for qubit generation by GC.

**Figure 8:**
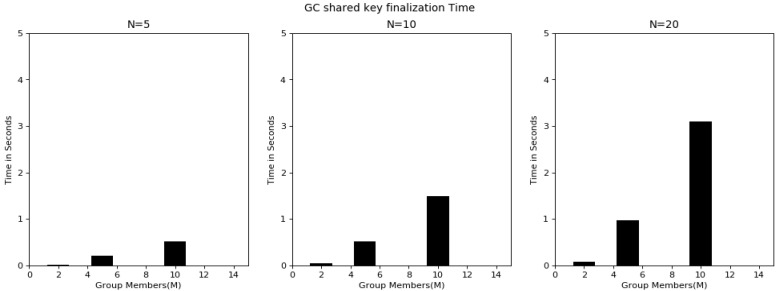
Time for shared key finalization with individual user through public discussion.

**Figure 9:**
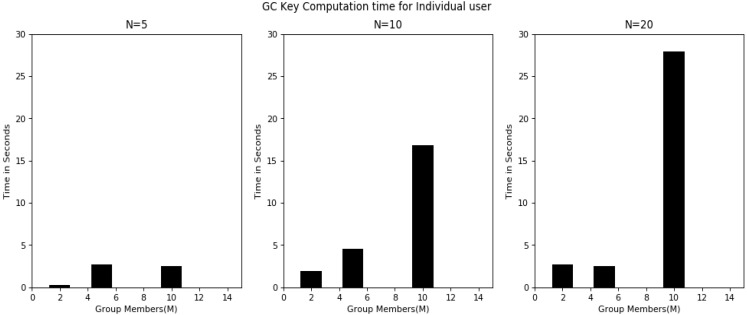
User partial key generation time by the GC.

**Figure 10:**
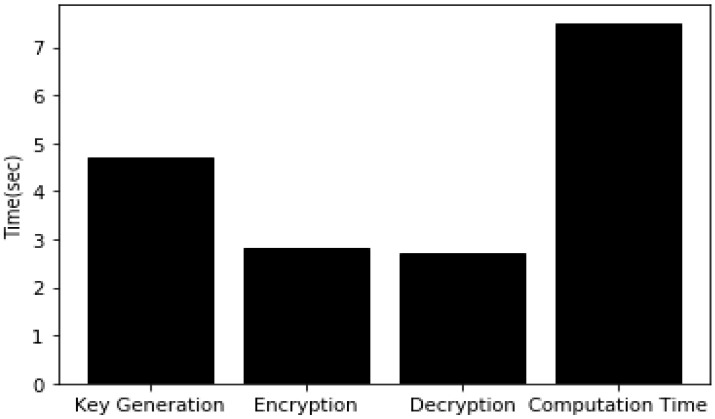
Time complexity of various operations for secure data processing in e-healthcare multi-agent system.

Overall, the experimentation was started using a group with 2 users, extended to 5 and 10 users and checked the functionality of the proposed algorithm. Figure 6 shows the key-sharing time between individual users and the GC by varying variable key lengths. The graph shows the key-sharing time for 5-, 10- and 20-bit lengths. Key-sharing time is the time used in quantum key transmission between user i and the GC. As the detailed process explains in Table 4, this was always constant irrespective of group users. However, the time increased linearly with respect to the increase in bit length, because, as the key length increased the time needed to guess the basis, as the public discussion in order to know the bases used by the user and final shred key derivation time also increased. Figure 7 shows the GC qubit generation time in the key-sharing process. This is the time the GC required to guess the bases upon receiving of the key from the user. The time in this process increased with an increase in group users. From Table 4, it can be observed that before finalization of the key between two users, there was a communication in public for sharing the basis. Figure 8 represents the time had spent in public conversation in order to finalize the key. Afterwards, GC finalized the key to individual user by performing the XOR operation on other n − 1 users. This computation time is presented in Figure 9. From the graph we can observe that with an increase in number of group members and the key length, computation time also increased. Figure 10 shows the secure communication time complexity between the data-collection agent and the department agent after collecting the doctor appointment dataset from the infrastructure layer. First, he derived the key with department agent using two-party quantum key agreement. Then, he encrypted the collected data using the key by doing the repeated XOR between the aggregated dataset and key. Next, the ciphered dataset was transferred onto department agent. The department agent collected the cipher dataset and then extracted the doctors appoint dataset using the key agreed with data-collection agent. Finally, the deciphered appointment dataset was stored onto the database. In the overall process, four phases were mainly involved: key agreement time complexity, encryption time complexity, decryption time complexity and overall computation time complexity. From the graph we can observe that more time was required for the key agreement phase than the encryption/decryption phase. Multicast and broadcast secure healthcare data sharing can be done using QDGKA—along with the encryption and decryption procedures specified in this paper.

## 7. Comparative Analysis of Protocols for Securing Multi-Agent-Based e-Healthcare System

The effectiveness of the proposed technique was compared with seven on hand MQKA protocols, namely LGMHW, HSXLFJY, WSH, SYW, SZWYZL and CM. A comparative analysis of MQKA protocols is presented below, and the precise comparative results are shown in Table 8. The features considered for performance comparison includes:

**Qubit efficiency (QE):** The qubit effectiveness is characterized as the proportion of the length of the final shared key (nc) foundation in the protocol to the total of the quantity of qubits utilized (q) and number of old style bits exchanged (b) for disentangling the message by barring the traditional correspondence utilized for checking the eavesdropping, Hence, *QE* = *nc*/(*q* + *b*). Concretely, to build up a L-bit final shared key in perfect state, every one of the included members ought to set up a sequence of *L* + *kL* photons, where k is detection rate. In the just one eavesdropping detection, every member will utilize *kL* photons in his/her groupings for checking spying. Since there are N members associated with the protocol proposed, the total quantity of the photons, which will be utilized in setting up a L-bit final shared key, is *N*(*L* + *kL*). Consequently, the qubit effectiveness of our protocol is, $QE=\frac{L}{N\left(L+kL\right)}$.

**Measuring Efficiency (ME)**: As the proposed protocol only requires detection of one eavesdropping, the amount of estimations essential in this protocol is relatively low. In particular, to set up an L-bit final shared key, in theory, each member is required to execute (*L* + *kL*) measurements. To be specific, *N*(*L* + *kL*) measurements are required in this whole process of the protocol. Consequently, the ME (the ratio of the length of final shared key to the quantity of the executed measurements) of the protocol is $ME=\frac{L}{N\left(L+kL\right)}=\frac{1}{N\left(1+k\right)}$.

**Unitary operation efficiency (UOE):** In view of the fact that the protocol’s security is mostly based on the unitary operations executed on the transmitted photons. Here, we compute the UOE as the ratio of the length of final shared key to the quantity of the executed unitary operations of the protocol. Concretely, to set up an L bit final shred key, every member is required to execute *N*(*L* + *kL*) unitary operations in theory. Specifically, $N^{2}\left(L+kL\right)$ unitary operations are required in total. Thus, UOE of our protocol is $UOE=\frac{L}{N^{2}\left(L+kL\right)}=\frac{1}{N^{2}\left(1+k\right)}$.

In addition, in the current MQKAP, after the members affirm that there exists no eavesdropping in the executing method of the protocol—every member straightforwardly utilizes the estimations after the effects of the rest of the quantum data transporters conclude a given binary string as his/her final key.

LGMHW protocol [52]: This protocol is in opposition to against participant attack and does not require entanglement; efficient with QE value $\frac{1}{N\left(N-1\right)}$ and UOE is zero.

HSXLFJY protocol [59]: This protocol is secured in opposition to participant attack and does not require entanglement; efficient with QE value $\frac{1}{\begin{array}{c} 2N^{2} \end{array}}$ and UOE is $\frac{1}{\begin{array}{c} N^{2} \end{array}}$.

WSH protocol [63]: This protocol is secured in opposition to participant attack and does not require entanglement; efficient with QE value $\frac{1}{2N\left(N-1\right)}$ and UOE is $\frac{1}{N\left(N-1\right)}$.

SYW protocol [58]: This protocol is not secured in opposition to participant attack and requires entanglement; efficient with QE value $\frac{2}{N\left(N+4\right)}$ and UOE is $\frac{2}{N\left(N-1\right)}$.

SZWYZL protocol [57]: This protocol is not secured in opposition to participant attack and requires entanglement; efficient with QE value $\frac{1}{N\left(2N+3\right)}$ and UOE is $\frac{2}{N\left(2N-1\right)}$.

CM protocol [60]: This protocol is secured in opposition to participant attack and requires entanglement; efficient with QE value $\frac{2}{N\left(N+1\right)}$ and UOE is $\frac{2}{N\left(N+1\right)}$.

HUA protocol [64]: This protocol is secured in opposition to participant attack and does not require entanglement; efficient with QE value $\frac{1}{2N}$ and UOE is $\frac{1}{\begin{array}{c} 2N^{2} \end{array}}$.

Proposed protocol: This protocol is secured in opposition to participant attack and requires entanglement; efficient with QE value $\frac{1}{2N}$ and UOE is $\frac{1}{\begin{array}{c} 2N^{2} \end{array}}$.

## 8. Conclusions and Future Work

This study has proposed a QDGKA protocol using QDH technique for securing e-healthcare MASs in the context of smart cities against quantum-based attacks. The security provided by the protocol is capable to resist the eavesdropping attacking from internal as well as external participants. We showed that our protocol is secure against participant attack and requires entanglement; efficient with QE value $\frac{1}{2N}$ and UOE is $\frac{1}{\begin{array}{c} 2N^{2} \end{array}}$. The security of the proposed solution is based on the unconditional security of the QDH. The results in the context of group key agreement among the users in the smart city show the potentiality of the proposed QDGKA in comparison with existing alternatives. Further, the computation complexity of the proposed work is evaluated on doctor’s appointment dataset and obtained satisfactory results in executing a multi-agent-based e-healthcare system in a smart city.

As a part of future work, we may further establish formal security model for this dynamic quantum group key agreement, thus council authorities trust these systems to be actually deployed in e-healthcare MASs of smart cities. We also plan to integrate the proposed approach to improve our system for improving mobility and quality of life of visually impaired people [65] for improving the security of this system.

## Figures and Tables

**Table 1:** List of acronyms of key concepts used in this article.

Notation	Description
QKD	Quantum key distribution
QKA	Quantum key agreement
MQKA	Multi-party QKA
QGKA	Quantum group key agreement protocol
DQGKA	dynamic quantum group key agreement protocol
MAS NJGK	Multi agent systems New join group key
NLGK	New leave group key
GC	Group controller
PGK	Previous group key
*m*	Number of bits exchanges between the nodes

**Table 2:** DQGKA computation and communication cost.

Protocol	Communication				Computation
DQGKA	Rounds	Messages	Unicast	Broadcast	XOR Operation
Initialize	2	*m* − 1	*m* − 1	0	2 *m*
Join	1	2	1	1	4
Leave	1	*m* − 2	*m* − 2	0	3

**Table 3:** Intercept and resend attack.

Quantum Key Transmission between User A, E and B										
User-A random bits	1	1	1	1	0	0	1	1	1	1
Random sending bases	X	X	+	+	+	X	X	+	X	X
Photons user-A sends	↘	↘	→	→	↑	↗	↘	→	↘	↘
E’s measuring basis	X	X	+	X	+	X	+	+	+	X
Polarization Eve measures and communicate	↘	↘	→	↘	→	↗	↘	→	↘	↘
B’s measuring basis	↘	↘	↘	→	→	↗	↘	→	↘	↘
Random received bases	X	X	X	+	+	X	+	X	+	X
**PUBLIC DISCUSSION**										
User-A says which bases were correct	√	√		√		√				√
**OUTCOME**										
Shared key between user-A and B	1	1		1		0				1

Basis used +0: ↑; +1: →; X0: ↗; X1:↘.

**Table 4:** Shared key calculated by all the other group users except GC.

Quantum Key Transmission between User-i and GC										
User-i random bits	1	1	1	1	0	0	1	1	1	1
Random sending bases	*X*	*X*	*+*	*+*	*+*	*X*	*X*	*+*	*X*	*X*
Photons user-i sends	↘	↘	→	→	↑	↗	↘	→	↘	↘
Random received bases	*X*	*X*	*X*	*X*	*+*	*X*	*+*	*+*	*+*	*X*
Bits GC has received	1	1	1	0	1	0	1	1	0	1
**PUBLIC DISCUSSION**										
GC reports the bases of received bits	*X*	*X*	*X*	*X*	*+*	*X*	*+*	*+*	*+*	*X*
Photons GC measured	↘	↘	↘	↗	→	↗	→	→	↑	↘
User-i says which bases were correct	*√*	*√*				*√*				*√*
**OUTCOME**										
Shared key between user-i and GC	1	1				0				1

Basis used: +0: ↑; +1: →; X0: ↗; X1: ↘.

**Table 5:** Key component established by GC for the respective members.

User	Key Index	Individual Members-Two Party Keys Established with GC $\left(K_{i}\right)$	Two Party Keys after Length Adjustment	Partial Key Components Established by GC for the Respective Members
User-2	$K_{2}$	1101	1101	$K_{3}⊕K_{4}⊕K_{5}$
User-3	$K_{3}$	101	0101	$K_{2}⊕K_{4}⊕K_{5}$
User-4	$K_{4}$	0100	0100	$K_{2}⊕K_{3}⊕K_{5}$
User-5	$K_{5}$	1111	1111	$K_{2}⊕K_{3}⊕K_{4}$

**Table 6:** Computation of final group key by all the users of e-healthcare.

User Number	Partial Key Component sent by GC $\left(PKey_{GCi}\right)$	Individual Two-Party Keys Established with GC $\left(K_{i}\right)$	Final GROUP KEY $Key_{i}=PKey_{GCi}⊕K_{i}$
User-2	0101	1101	1000
User-3	1101	0101	1000
User-4	1100	0100	1000
User-5	0111	1111	1000

**Table 7:** Qiskit functions.

Function Name	Description
Quantum Register (n, name=‘qr’)	Used to store one qubit bit
Classical Register (n, name=‘cr’)	For storing the output of the measurement
Quantum Circuit (qr, cr, name=‘Alice’)	Collections of quantum gates interconnected by quantum wires
np.random.randint (0, high=2n)	Generate a random number between 0 to 2n
np.binary_repr (alice_key, n)	Returns the binary equivalent of the given number n as a string
BasicAer.get_backend (‘qasm_simulator’)	Simulates the circuit in the backend
execute (bob, backend = backend, shots = 1).result()	Executes the circuit created using qsam simulator in the backend

**Table 8:** Comparative analysis of multiparty QKA (MQKA) protocols.

MQKA Protocol	QE	UO	UOE	SAP	KCC	Entanglement Required
LGMHW [52]	$\frac{1}{N\left(N-1\right)}$	no	0	secure	no	no
HSXLFJY [59]	$\frac{1}{\begin{array}{c} 2N^{2} \end{array}}$	yes	$\frac{1}{\begin{array}{c} N^{2} \end{array}}$	secure	no	no
WSH [63]	$\frac{1}{2N\left(N-1\right)}$	yes	$\frac{1}{N\left(N-1\right)}$	secure	no	no
SYW [58]	$\frac{2}{N\left(N+4\right)}$	yes	$\frac{2}{N\left(N-1\right)}$	insecure	no	yes
SZWYZL [57]	$\frac{1}{N\left(2N+3\right)}$	yes	$\frac{2}{N\left(2N-1\right)}$	insecure	no	yes
CM [60]	$\frac{1}{N\left(N+1\right)}$	yes	$\frac{2}{N\left(N+1\right)}$	secure	no	yes
HUA [64]	$\frac{1}{2N}$	yes	$\frac{1}{\begin{array}{c} 2N^{2} \end{array}}$	secure	yes	no
Proposed	$\frac{1}{2N}$	yes	$\frac{1}{\begin{array}{c} 2N^{2} \end{array}}$	secure	yes	yes

*N* = number of participants.
